# Adolescents' cross-cultural aesthetic preferences are shaped by cultural background, art exposure, and developmental psychological traits

**DOI:** 10.3389/fpsyg.2026.1774986

**Published:** 2026-06-18

**Authors:** Hongmei Wang, Xiaoou He

**Affiliations:** 1 School of Art and Design, University of Jinan, Jinan, China; 2 School of Art of Soochow University, Suzhou, China

**Keywords:** adolescent development, aesthetic preferences, art exposure, cognitive flexibility, cross-cultural aesthetics, openness to experience

## Abstract

**Background:**

Adolescence is a critical developmental period for cultural learning and the formation of aesthetic preferences. As adolescents increasingly encounter visual art from diverse cultural traditions through educational and digital contexts, understanding how cultural background, experiential factors, and individual psychological traits jointly shape cross-cultural aesthetic preferences is essential. However, research in cross-cultural aesthetics has largely focused on adults, leaving adolescent development relatively underexplored.

**Methods:**

This study examined visual art preferences among 400 adolescents aged 14–18 from Chinese and Western cultural backgrounds. Participants evaluated artworks from Chinese and Western traditions and completed measures assessing exposure to global art forms, openness to experience, and cognitive flexibility. Hierarchical regression analyses were conducted to test the mediating role of art exposure and the moderating effects of psychological traits on the relationship between cultural background and aesthetic preferences.

**Results:**

Cultural background significantly predicted adolescents' aesthetic preferences, with a general tendency to prefer artworks from one's own cultural tradition. Exposure to global art forms partially mediated this relationship, such that greater cross-cultural art engagement was associated with weaker culturally congruent preferences. In addition, openness to experience and cognitive flexibility moderated cultural effects: adolescents higher in these traits showed reduced cultural bias in their aesthetic judgments. Models integrating cultural, experiential, and psychological factors explained substantially more variance in aesthetic preferences than models based on cultural background alone.

**Conclusions:**

These findings suggest that adolescents' cross-cultural aesthetic preferences are shaped by the interaction of cultural background, learning-related art exposure, and individual developmental characteristics. Aesthetic preferences during adolescence are not fixed outcomes of cultural membership but remain responsive to experience and psychological differences. This study advances a developmentally informed understanding of cross-cultural aesthetic learning in adolescence.

## 1 Introduction

In an increasingly globalized and digitized world, adolescents are growing up in cultural environments characterized by unprecedented exposure to diverse visual stimuli and artistic traditions. Visual arts, facilitated by broader media environments and global communication networks, now routinely transcend national boundaries and cultural barriers, shaping the everyday perceptual and learning experiences of young people across the globe (Yunta, 2011; Zijlmans and Van Damme, 2008). For adolescents aged 14–18—a critical developmental period marked by rapid cognitive, emotional, and social change—engagement with visual art is not merely a matter of aesthetic enjoyment, but an important context for identity formation, cultural learning, and emotional development (Pelowski et al., 2016). From a human developmental psychology perspective, adolescence represents a sensitive window in which individuals' cognitive styles, emotional responsiveness, and openness to cultural difference undergo substantial reorganization. During this stage, advances in abstract thinking, perspective-taking, and self-reflection enable adolescents to process visual information in increasingly complex ways, while heightened emotional sensitivity intensifies their responses to aesthetic and symbolic content (Jacobsen, 2010). As a result, adolescents' aesthetic preferences and emotional engagement with art are particularly susceptible to cultural influences as well as to broader learning experiences associated with increasing exposure to culturally diverse visual content in contemporary media environments (Windhager and Mayr, 2022).

Chinese and Western visual art traditions, grounded in distinct philosophical, historical, and cultural frameworks, offer a valuable lens for examining how cultural contexts shape adolescents' developing perceptual and interpretive processes (Szubielska et al., 2025). Chinese visual art traditions often emphasize harmony, balance, contextual relationships, and spiritual meanings, whereas Western traditions have historically foregrounded realism, individual expression, and emotional intensity (Daryanavard et al., 2025). Developmental research suggests that such cultural differences are closely linked to broader cognitive and attentional patterns acquired through socialization, education, and everyday cultural practices (Park and Huang, 2010; Van Geert et al., 2024). Recent empirical research in cross-cultural psychology and neuroaesthetics has further demonstrated that aesthetic perception involves dynamic interactions among cultural learning, attentional processing, emotional evaluation, and neural responsiveness to visual stimuli (Feng et al., 2022; Wei et al., 2025). Contemporary neuroaesthetic perspectives suggest that aesthetic experiences are shaped not only by perceptual characteristics of artworks, but also by culturally informed cognitive schemas and individual differences in exploratory processing, emotional sensitivity, and cognitive flexibility (Meng and Liang, 2022; Wei et al., 2025). Emerging developmental evidence further indicates that adolescents may exhibit heightened responsiveness to culturally unfamiliar visual information due to ongoing maturation of executive functioning, emotional regulation, and social-cognitive processing systems (Telzer et al., 2025). At the same time, it is important to recognize that both “Chinese” and “Western” cultural backgrounds encompass substantial internal diversity shaped by national, ethnic, linguistic, educational, and social differences. Accordingly, the present study does not treat these categories as culturally homogeneous or essentialized groups, but rather as broad developmental cultural environments relevant to adolescents' dominant socialization experiences. For adolescents, whose perceptual habits and aesthetic values are still being actively shaped, these contrasting artistic traditions may exert particularly strong and malleable influences on how artworks are perceived, interpreted, and emotionally evaluated. Despite growing interest in cross-cultural aesthetics, existing research has largely focused on adult populations, leaving adolescent audiences underrepresented in empirical investigations (Jacobsen, 2010). This omission is notable, as adolescence is a formative period for the development of aesthetic sensitivity, cultural values, and learning preferences. Moreover, much of the current literature examines either cultural background or individual psychological traits in isolation, without sufficiently addressing how these factors interact with adolescents' broader exposure to culturally diverse artistic traditions in contemporary media contexts (Ayele et al., 2025; van Paasschen et al., 2015). Although prior studies have documented cultural differences in visual attention and perception (Chua et al., 2005; Heesen et al., 2024), relatively limited research has examined how broader contemporary media environments may relate to adolescents' exposure to culturally diverse visual traditions and aesthetic learning opportunities (Budge, 2013). More recent empirical studies using eye-tracking, neurocognitive, and experimental paradigms have also demonstrated that cultural background may influence attentional allocation, perceptual organization, and emotional responses during aesthetic evaluation processes (Chen et al., 2023; Guo et al., 2024). These findings support the view that cross-cultural aesthetic preferences reflect not only learned cultural values, but also broader perceptual-cognitive processing differences associated with developmental and sociocultural experiences. Furthermore, recent adolescent visual cognition research suggests that perceptual flexibility and evaluative adaptability continue to develop throughout adolescence, potentially increasing adolescents' responsiveness to culturally diverse artistic stimuli (Parr et al., 2024).

The rapid expansion of global communication and media environments has also created new opportunities for developmental and cross-cultural research. Adolescents are increasingly exposed to culturally diverse visual content through contemporary media environments and globalized visual communication contexts, which may broaden opportunities for cross-cultural artistic exposure and aesthetic learning (Johanson et al., 2024; Yap et al., 2024). Although the present study does not employ large-scale behavioral analytics, computational modeling, or direct measures of digital engagement, contemporary media environments nonetheless provide an important contextual background for understanding adolescents' exposure to culturally diverse visual content. Previous research has suggested that broader media environments may influence aesthetic learning and cross-cultural exposure patterns during adolescence (Mobo et al., 2025). However, the integration of broader cultural exposure research with established developmental and cultural psychological theories remains limited, posing challenges for interpreting how contemporary visual environments may contribute to adolescents' aesthetic development (Kitchin, 2014; Lauret et al., 2010; Klinke, 2014). Accordingly, digital and media-related contexts are discussed in the present study primarily as broader sociocultural environments shaping adolescents' exposure opportunities rather than as directly measured explanatory constructs within the empirical model. Against this backdrop, the present study aims to advance developmental and cross-cultural aesthetic research by examining how cultural background influences aesthetic preferences in visual arts among adolescents aged 14–18, with particular attention to the potential indirect role of exposure to global art forms and the moderating effects of individual psychological traits, including openness to experience and cognitive flexibility (Fayn et al., 2015). By focusing on adolescents, this research highlights a developmental stage in which learning, identity formation, and cultural sensitivity are especially dynamic. Using a regression-based multivariable analytical framework, the study seeks to examine how cultural background, individual dispositions, and exposure to diverse artistic traditions jointly relate to adolescents' cross-cultural aesthetic preferences within broader contemporary sociocultural contexts (Klinenberg and Benzecry, 2005).

The present study contributes to the literature in several important ways. First, unlike prior cross-cultural aesthetic research that has primarily focused on adult populations or examined cultural background and psychological characteristics as relatively independent predictors of aesthetic preference, the present study adopts a developmental-integrative perspective on adolescents' cross-cultural aesthetic formation. Specifically, adolescents' aesthetic preferences are conceptualized as dynamic developmental outcomes associated with the combined influence of cultural socialization, exposure to global art forms, and individual psychological traits. Second, this research extends existing cross-cultural aesthetic theory by integrating developmental psychology with cultural and experiential approaches to art appreciation. Within the proposed framework, cultural background is treated as a foundational socialization context, while exposure to global art forms is conceptualized as a learning-related mediating mechanism through which broader cultural environments may be associated with adolescents' aesthetic preferences. In contrast, openness to experience and cognitive flexibility are conceptualized as developmental psychological dispositions that influence how adolescents interpret, evaluate, and respond to culturally unfamiliar visual stimuli. Accordingly, the mediating and moderating mechanisms are treated as theoretically distinct but complementary developmental processes rather than competing explanations. In doing so, the study moves beyond static cultural comparison models and provides a more process-oriented understanding of adolescents' cross-cultural aesthetic development. Third, the study highlights adolescence as a particularly important developmental period for cross-cultural aesthetic learning. Because adolescence is characterized by heightened cognitive plasticity, identity exploration, and increased sensitivity to social and cultural experiences, adolescents' aesthetic preferences may remain more malleable than those observed in adulthood. The findings therefore contribute to a more dynamic understanding of cross-cultural aesthetic development within increasingly globalized cultural contexts. Practically, the findings have implications for art education, intercultural communication, and cultural policy initiatives aimed at fostering inclusive and globally informed learning environments for young people. By clarifying the developmental processes through which adolescents engage with culturally diverse visual art, this research provides evidence-based insights for designing educational practices and cultural programs that support creativity, intercultural understanding, and aesthetic openness in an increasingly interconnected world.

## 2 Theoretical foundation and research hypotheses

### 2.1 Cultural influences on adolescents' aesthetic preferences and perceptual development

Aesthetic preferences—defined as subjective evaluations of beauty and pleasantness in response to visual stimuli—are not static traits but developmental outcomes shaped through continuous interaction between cultural, social, and individual factors (Vessel et al., 2018). From a developmental psychology perspective, these preferences are progressively constructed across childhood and adolescence as individuals acquire culturally patterned perceptual habits, emotional responses, and interpretive frameworks (Palmer et al., 2013). During adolescence in particular, advances in abstract reasoning, perspective-taking, and emotional sensitivity render aesthetic judgments especially responsive to cultural influences and learning experiences (Mastandrea et al., 2019). A substantial body of cross-cultural research has demonstrated that cultural background plays a critical role in shaping how individuals attend to, interpret, and emotionally engage with visual information. For instance, Masuda et al. (2008) found that East Asian participants tended to allocate greater attention to contextual relationships within visual scenes, whereas Western participants focused more on salient focal objects and their attributes (Masuda et al., 2008). These perceptual tendencies are closely aligned with broader cultural values: collectivistic orientations prevalent in many Chinese cultures foster holistic modes of perception, while individualistic orientations common in Western cultures promote analytic and object-focused processing (Vignoles et al., 2024).

Importantly, such culturally shaped perceptual styles are not only reflected in adult cognition but are actively internalized during adolescence through formal education, media exposure, and everyday cultural practices. These differences manifest not only in audience responses but also in the formal characteristics of artistic traditions themselves (Senzaki et al., 2014). Traditional Chinese landscape painting, for example, emphasizes harmony with nature, spatial continuity, and relational balance among elements (Xiaoxuan, 2022), whereas Western Renaissance art foregrounds individual subjects, realism, and emotional expressiveness (Kemp, 1990). For adolescents, repeated exposure to these culturally embedded artistic conventions contributes to the formation of stable aesthetic schemas that guide perception and evaluation. The philosophical and religious foundations underlying these artistic traditions further shape adolescents' aesthetic development. Chinese visual arts frequently draw on Confucian, Taoist, and Buddhist philosophies, emphasizing impermanence, balance, and interconnectedness (Lomas, 2016). In contrast, Western art reflects the influence of Christianity and Renaissance humanism, privileging individual agency, emotional depth, and exploration of the natural world (Van Geert et al., 2024). During adolescence—a period marked by identity exploration and meaning-making—such value-laden aesthetic systems may exert particularly strong influences on emotional engagement with art.

However, in contemporary digital environments, adolescents are increasingly exposed to diverse global art forms that transcend traditional cultural boundaries (Belting et al., 2013). The widespread popularity of anime—an art form rooted in Japanese culture—among Western adolescents exemplifies the growing potential for cross-cultural aesthetic learning (Denison, 2015). Similarly, the global circulation and appreciation of Western impressionist art among Chinese youth illustrates the reciprocal nature of cultural exchange in the digital age (Yu, 2022). These patterns suggest that exposure to global art forms may play a mediating role in the relationship between cultural background and aesthetic preferences, particularly during adolescence, when learning and perceptual systems remain developmentally plastic (Swami, 2013). In the present framework, exposure to global art forms is conceptualized as an experiential learning mechanism associated with adolescents' opportunities for cross-cultural aesthetic engagement, rather than as a stable individual psychological disposition.

H1: Cultural background (Chinese vs. Western) has a significant effect on adolescents' aesthetic preferences in visual arts, with exposure to global art forms mediating this relationship.

### 2.2 Individual psychological traits in adolescence: openness, cognitive flexibility, and aesthetic development

In addition to cultural influences, individual psychological traits play a crucial role in shaping aesthetic preferences and emotional responses to art, particularly during adolescence, when personality structures and cognitive control systems continue to mature (Fayn et al., 2015). Among these traits, openness to experience and cognitive flexibility have been identified as especially relevant for understanding individual differences in aesthetic engagement (Leder et al., 2018). Openness to experience, a core dimension of the Big Five personality model, refers to an individual's tendency toward curiosity, imagination, and receptivity to novel ideas and experiences (Kaufman et al., 2016). Developmental research suggests that openness becomes increasingly differentiated during adolescence, as individuals gain greater autonomy in exploration and learning. Adolescents high in openness tend to display broader aesthetic interests, greater tolerance for ambiguity, and a stronger appreciation for unconventional or abstract art forms (Swami, 2013). Openness has also been associated with heightened emotional responsiveness to art and more intense aesthetic experiences (Silvia, 2011), indicating its importance for cross-cultural aesthetic appreciation. Cognitive flexibility, by contrast, refers to the capacity to adapt thinking and behavior in response to changing situational demands and multiple perspectives (Tello-Ramos et al., 2019). During adolescence, the development of executive functions supports increasing flexibility in reasoning and interpretation. In the context of art appreciation, cognitive flexibility enables adolescents to shift between different cultural frames of reference, integrate unfamiliar symbolic meanings, and derive aesthetic value from complex or ambiguous visual stimuli (Pelowski et al., 2016). Empirical studies have shown that cognitive flexibility is positively associated with aesthetic sensitivity, creative thinking, and the ability to find meaning in non-representational or culturally unfamiliar artworks (De Dreu et al., 2012).

Taken together, these traits may buffer the influence of culturally ingrained aesthetic biases by facilitating openness to novelty and adaptive interpretation. Adolescents high in openness to experience or cognitive flexibility may therefore be better equipped to engage with art from cultures other than their own, showing reduced cultural bias in aesthetic judgment. Accordingly, we propose the following hypotheses:

H2: Openness to experience moderates the relationship between cultural background and adolescents' aesthetic preferences, such that individuals high in openness show smaller cultural differences in preferences than those low in openness.

H3: Cognitive flexibility moderates the relationship between cultural background and adolescents' aesthetic preferences, such that individuals high in cognitive flexibility show smaller cultural differences in preferences than those low in cognitive flexibility.

### 2.3 Integrative multivariable approaches to studying adolescents' aesthetic development in digital contexts

Most prior research on aesthetic preferences and audience psychology has relied on small-scale laboratory experiments or self-report surveys, which limit ecological validity and may inadequately capture adolescents' everyday interactions with visual art (Locher, 2012). In contrast, contemporary adolescents engage with art increasingly through digital platforms, social media, and online visual environments, creating new opportunities to study aesthetic development in more ecologically relevant contexts (Yap et al., 2024). Although the present study does not employ large-scale behavioral datasets or computational analytics, digital media environments provide an important contextual framework for understanding adolescents' exposure to culturally diverse visual content and artistic experiences.

Integrative multivariable analytical approaches offer several advantages for investigating adolescents' aesthetic preferences across cultural contexts. First, they enable researchers to examine how multiple developmental, cultural, and psychological factors jointly relate to adolescents' aesthetic judgments rather than treating aesthetic preference as the outcome of a single cultural variable. Such approaches are particularly valuable in developmental research, where adolescents' preferences are shaped simultaneously by cultural learning, educational experiences, peer influence, and exposure to diverse media environments (Waller and Fawcett, 2013).

Second, multivariable analytical frameworks allow researchers to incorporate contextual and individual-difference variables, including exposure to global art forms, openness to experience, and cognitive flexibility. This is especially relevant for adolescence, a developmental period characterized by rapid cognitive, emotional, and social change (Philip Chen and Zhang, 2014). Examining these factors simultaneously may provide a more comprehensive understanding of how adolescents respond to culturally familiar and unfamiliar artworks.

Third, prior digital culture research suggests that adolescents' aesthetic experiences are increasingly shaped by online visual environments and cross-cultural media exposure (Manovich et al., 2017). While the present study uses regression-based analyses rather than advanced computational modeling techniques, the conceptual framework was informed by broader interdisciplinary discussions concerning digital visual culture, cultural exposure, and adolescent learning environments. This perspective supports the examination of complex associations among cultural background, psychological traits, and exposure to global art forms during adolescence.

**Figure 1:**
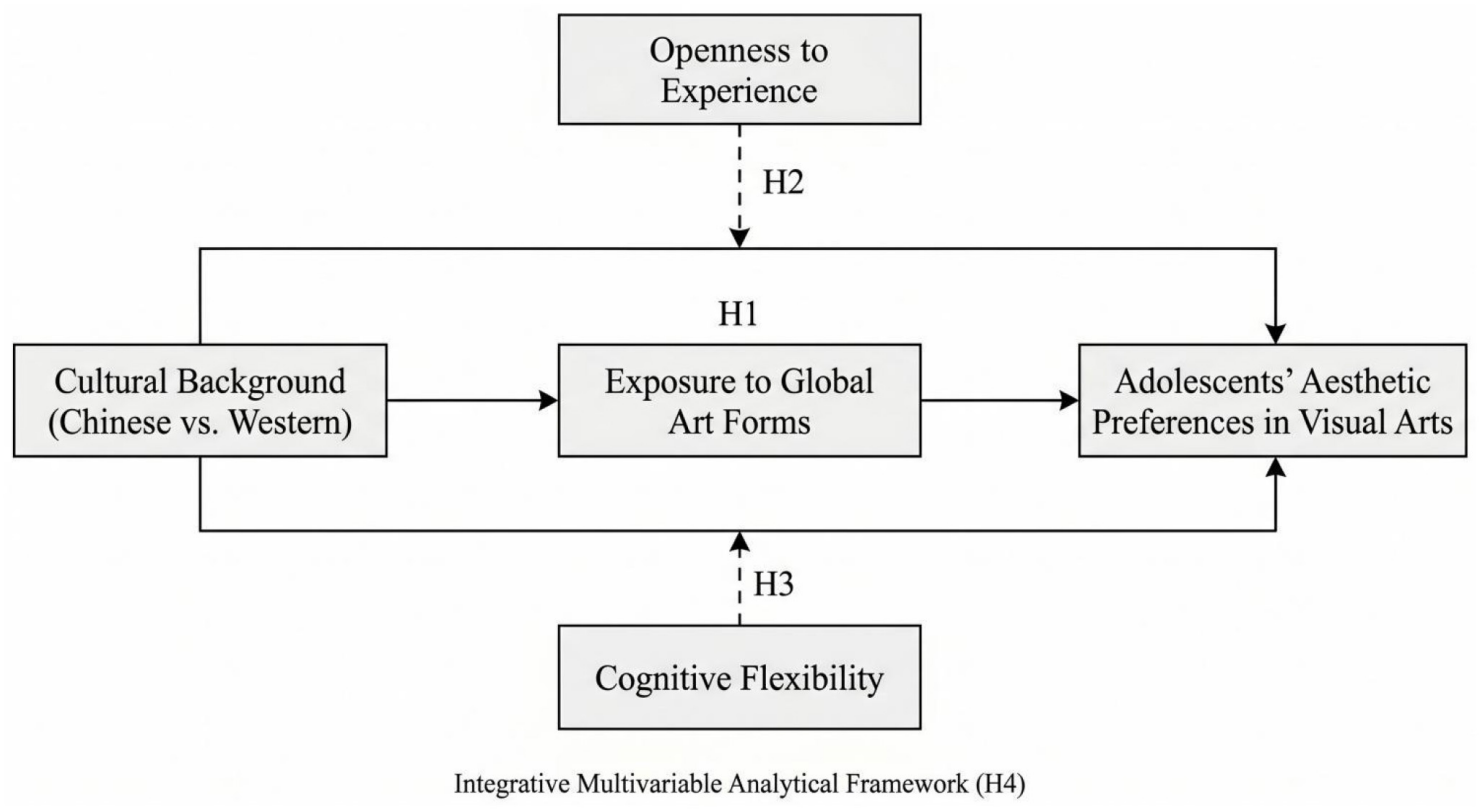
Theoretical framework of the study.

Despite these advantages, research on adolescent aesthetic development in digital environments continues to face important methodological and ethical challenges, including measurement validity, cultural heterogeneity, privacy protection for minors, and the integration of developmental theory with emerging digital-context research. Accordingly, the present study should be understood as an exploratory regression-based multivariable analysis rather than a computational or big-data modeling study. Nevertheless, when theoretically grounded and developmentally informed, multivariable analytical approaches can contribute meaningfully to understanding adolescents' aesthetic development in culturally diverse contemporary environments. Based on these considerations, we propose the following hypothesis:

H4: Integrative multivariable analytical approaches that consider cultural background, exposure to global art forms, and individual psychological traits together may provide a more comprehensive understanding of adolescents' aesthetic preferences than single-factor analytical approaches (Figure 1).

## 3 Data and method

### 3.1 Participants and data collection procedure

#### 3.1.1 Participants

The participants in this study were adolescents aged 14–18 years, a developmental period characterized by rapid cognitive, emotional, and socio-cultural change. This age range was selected to capture key stages of adolescent aesthetic development, including middle to late adolescence (Sawyer et al., 2018; Rocha et al., 2020; Rubin et al., 2024; Demaria et al., 2024). The final sample included both senior high school students (Grades 10–12) and first-year university students (Grade 13), reflecting variation in educational context while remaining within the targeted developmental window.

Participants were recruited from secondary schools, international educational programs, and comprehensive universities in China. The sample included adolescents from both Chinese cultural backgrounds and Western cultural backgrounds. Adolescents classified as having Chinese cultural backgrounds were primarily Chinese students who had received long-term education within Chinese cultural and educational environments. Adolescents classified as having Western cultural backgrounds included international students and adolescents from predominantly North American and European family cultural backgrounds who were studying in international schools or university preparatory programs in China at the time of data collection.

Cultural background classification was determined based on participants' self-reported primary cultural environment, including family cultural background, long-term residence, language use in daily life, educational experience, and dominant cultural exposure during upbringing. Participants who reported mixed cultural experiences were asked to identify the cultural context that had the strongest influence on their daily life and value formation. The present study adopted a developmental cultural socialization perspective, emphasizing adolescents' dominant cultural learning environments during formative developmental stages rather than nationality alone.

Although the use of broad cultural categories necessarily simplifies within-group cultural diversity, this classification approach was intended to capture differences in dominant developmental cultural environments relevant to adolescents' aesthetic learning and cross-cultural artistic exposure.

Inclusion criteria required that participants (a) fell within the age range of 14–18 years, (b) were currently enrolled in secondary or tertiary education, and (c) had sufficient language proficiency to comprehend the questionnaire items. Students with identified visual impairments that could affect image perception were excluded from the study.

#### 3.1.2 Data collection procedure

Data were collected using a structured, self-administered online questionnaire designed specifically for adolescent participants. Prior to formal data collection, the questionnaire underwent a two-stage pilot testing process to ensure age appropriateness, clarity, and measurement reliability.

In the first stage, the initial version of the questionnaire was reviewed by a panel of experts, including developmental psychologists, art education researchers, and secondary school teachers with experience in adolescent education. This expert review focused on the developmental suitability of the wording, the cultural neutrality of the instructions, and the appropriateness of the visual stimuli for adolescents.

In the second stage, a pilot study was conducted with a separate sample of 52 adolescents (aged 14–18) who were not included in the final dataset. Feedback from the pilot participants was used to refine item wording, adjust questionnaire length, and confirm that all visual materials were clearly recognizable and comprehensible. Results from the pilot study indicated acceptable internal consistency for all multi-item scales, with Cronbach's alpha values ranging from 0.78 to 0.86 across key measures.

Following pilot testing, the formal data collection was carried out over a four-week period. A total of 450 questionnaires were distributed to eligible adolescent participants through participating schools and universities. Of these, 420 questionnaires were returned, resulting in an initial response rate of 93.3%.

To improve data quality, several screening procedures were implemented before statistical analysis. Questionnaires were excluded if they met one or more of the following criteria: (a) more than 10% missing responses across questionnaire items; (b) identical responses across all Likert-scale questions, indicating patterned or inattentive responding; (c) failure on embedded attention-check items; or (d) completion times shorter than one-third of the median survey completion time. In addition, duplicate submissions identified through system records were removed. After the screening process, 400 questionnaires were retained as valid for final analysis, yielding an effective response rate of 88.9%.

#### 3.1.3 Survey administration

The finalized questionnaire was administered online using a secure survey platform. For high school students under the age of 18, questionnaires were distributed through school administrators and teachers during designated class periods or supervised study sessions. First-year university students completed the survey either during scheduled academic activities or independently through institutional learning platforms.

Before accessing the questionnaire link, eligible participants received a brief written explanation describing the purpose of the study, estimated completion time, confidentiality procedures, and voluntary nature of participation. For participants under 18 years old, access to the online questionnaire was provided only after parental or legal guardian consent had been obtained through the participating schools or institutional communication channels.

To ensure data quality and consistency, all participants completed the questionnaire individually under standardized instructions. The average completion time was approximately 15–20 min. No identifying personal information was collected, and all responses were recorded anonymously.

#### 3.1.4 Ethical considerations and informed consent

The study was conducted in accordance with the ethical standards outlined in the Declaration of Helsinki and was approved by the Institutional Review Board of the School of Art at Soochow University.

Given that the study involved minors, a two-tier informed consent process was implemented. Written informed consent was obtained from parents or legal guardians of all participants under the age of 18 prior to data collection. In addition, adolescents themselves provided written informed assent after receiving a clear explanation of the study's purpose, procedures, potential risks, and their right to withdraw at any time without penalty. For first-year university students aged 18, informed consent was obtained directly from the participants.

For adolescent participants under the age of 18, parental consent forms were distributed electronically through participating schools and institutional communication systems before the survey period began. Parents or legal guardians reviewed the study information sheet and provided written electronic consent before adolescents were permitted to access the online questionnaire. At the beginning of the survey, adolescent participants additionally completed an electronic assent form confirming that they understood the study procedures and agreed to participate voluntarily. Participants could discontinue participation at any stage without academic or personal consequences.

All participants and parents were informed that the study involved viewing artworks and completing a questionnaire, that participation was voluntary, and that data would be used solely for academic research purposes.

#### 3.1.5 Research personnel and data quality control

The data collection process was conducted by a trained research team composed of researchers with formal backgrounds in psychology, art education, and developmental research methods. All research assistants received standardized training in ethical research practices, adolescent communication, and data confidentiality prior to data collection.

To ensure data quality, multiple screening procedures were applied. Responses with excessive missing data, unrealistically short completion times, or patterned responding were excluded from the final analysis. Only questionnaires that met predefined quality criteria were retained for statistical analysis.

In addition, survey administration was supervised by designated school coordinators or research assistants to ensure that participants completed the questionnaire independently and without external interference. Data were stored on password-protected devices accessible only to the research team. Prior to analysis, all datasets were anonymized and checked for coding accuracy and response consistency.

### 3.2 Variable

#### 3.2.1 Independent variable: cultural background in adolescence

Cultural background was operationalized as a categorical independent variable distinguishing adolescents' primary cultural socialization context as either Chinese cultural background or Western cultural background. The classification was based on participants' long-term cultural environment and educational experience during adolescence rather than nationality alone (Jefferson et al., 2023; Baumert et al., 2024). The present study adopted a developmental cultural socialization perspective, emphasizing adolescents' dominant cultural learning and value environments during formative developmental stages rather than treating cultural background as a fixed or homogeneous identity category.

To determine cultural background, participants completed demographic questions regarding: (a) long-term place of residence, (b) primary language used in daily life and education, (c) family cultural background, (d) type of school attended, and (e) dominant cultural environment during upbringing. Example questionnaire items included: “Which cultural environment has had the greatest influence on your daily life and values?” and “Which language do you primarily use in your everyday communication and education?” To reduce classification ambiguity, cultural background classification was determined using multiple indicators jointly rather than relying on a single self-report item. Participants' long-term residence, primary educational environment, dominant daily language, family cultural background, and self-identified primary cultural environment were considered together during classification.

Participants classified into the Chinese cultural background group were primarily Chinese adolescents who had received long-term education within Chinese schools and had grown up in Chinese cultural and social environments. Participants classified into the Western cultural background group included international students and adolescents from predominantly North American and European family cultural backgrounds who were enrolled in international schools or university preparatory programs in China during the period of data collection.

For participants reporting mixed cultural experiences, classification was based on the cultural environment that participants identified as having the strongest influence on their daily life, educational experience, and value formation during adolescence. More specifically, mixed-cultural participants were categorized according to the cultural environment that demonstrated the greatest overall consistency across the multiple classification indicators described above. Priority was given to adolescents' dominant educational environment and long-term developmental cultural exposure during adolescence.

To improve classification consistency, participant classifications were independently reviewed by two trained researchers based on the predefined classification criteria. Cases involving ambiguity or mixed-cultural backgrounds were discussed jointly until consensus was reached.

Although the use of broad cultural categories necessarily simplifies within-group diversity, the present classification approach was intended to capture differences in dominant developmental cultural environments relevant to adolescents' aesthetic learning, cultural exposure, and cross-cultural artistic experience rather than to imply culturally homogeneous groups. We acknowledge that considerable heterogeneity exists within both Chinese and Western cultural categories. Adolescents' aesthetic preferences may also be shaped by multiple intersecting factors, including ethnicity, bilingual or multilingual experiences, transnational educational exposure, regional cultural practices, and varying degrees of engagement with global media environments. Therefore, the present classification should be interpreted as reflecting adolescents' dominant developmental cultural socialization contexts rather than fixed or essentialized cultural identities.

#### 3.2.2 Dependent variable: adolescents' aesthetic preferences in visual arts

Adolescents' aesthetic preferences served as the dependent variable and were assessed through participants' evaluative responses to a set of visual artworks drawn from both Chinese and Western artistic traditions. The stimulus set included a balanced selection of paintings and visual compositions differing in stylistic features, compositional principles, and symbolic content, while remaining appropriate for adolescent viewers. A total of 24 visual artworks were included in the final stimulus set, consisting of 12 Chinese artworks and 12 Western artworks. The Chinese artworks primarily represented culturally recognizable traditional Chinese landscape and ink painting traditions characterized by contextual balance, symbolic imagery, and spatial harmony, whereas the Western artworks included examples from Western classical, realist, and Impressionist traditions emphasizing realism, perspective, and individual expression. For each artwork, participants rated their aesthetic appreciation using a 5-point Likert scale ranging from 1 (“Not appealing at all”) to 5 (“Extremely appealing”). These ratings were intended to capture adolescents' immediate affective and evaluative responses rather than formal art-historical judgments or technical expertise.

The artwork selection process was guided by several criteria. First, artworks were selected to represent culturally recognizable visual traditions while avoiding highly specialized or professionally technical artworks that might require advanced art-historical knowledge. Second, artworks containing extreme emotional, political, religious, or potentially disturbing content were excluded to ensure age appropriateness for adolescent participants. Third, the selected artworks varied in composition, color style, and visual complexity while remaining broadly comparable across cultural categories.

To strengthen the validity of stimulus categorization, all artworks were independently reviewed by three researchers with backgrounds in art education and cross-cultural visual studies. The reviewers evaluated the cultural representativeness and stylistic appropriateness of each artwork for the intended Chinese or Western visual art category. Only artworks reaching full consensus regarding cultural classification were retained in the final stimulus set.

Prior to formal data collection, the artwork stimuli were pre-tested with an independent group of adolescents to ensure cultural recognizability, age appropriateness, and sufficient variability in aesthetic appeal. Pilot participants were additionally asked to evaluate the familiarity and comprehensibility of the artworks. Highly recognizable or globally overexposed artworks were excluded from the final stimulus set in order to reduce familiarity bias and minimize the influence of prior exposure on adolescents' aesthetic evaluations. Mean preference scores were computed separately for Chinese and Western artworks, allowing for both overall preference assessment and culturally specific analyses.

Representative examples of the visual artworks used in the study are provided in the Supplementary material. The Supplementary materials include four representative stimulus examples illustrating the types of Chinese and Western artworks presented during the experiment. Prior to administration, all images were standardized in terms of size, resolution, and presentation format to ensure consistency across visual stimuli. In addition, the presentation order of the artworks was randomized across participants to minimize potential order effects and response fatigue. All images were displayed using the same digital presentation interface and viewing duration conditions.

#### 3.2.3 Mediating variable: exposure to global art forms during adolescence

Exposure to Global Art Forms was included as a mediating variable reflecting adolescents' cumulative engagement with visual art from diverse cultural traditions. Given adolescents' increasing reliance on digital media as a learning and exploration tool, this construct incorporated both offline and online modes of art exposure. Participants reported the frequency with which they: visited art exhibitions or museums (locally or internationally), engaged with visual art through digital platforms (e.g., online galleries, social media, educational art websites), encountered or discussed artworks from other cultures in school-based or informal learning contexts. Responses were recorded on a Likert scale ranging from “Never” to “Very frequently.” Item scores were aggregated to form a composite index of global art exposure, with higher scores indicating broader and more frequent cross-cultural art engagement. This variable operationalizes adolescents' opportunities for aesthetic learning beyond their primary cultural environment.

#### 3.2.4 Moderating variables: psychological traits in adolescence

Openness to Experience: Openness to Experience was assessed using a short, adolescent-appropriate version of the Big Five Inventory. The selected items focused on curiosity, imagination, and willingness to engage with novel ideas and experiences, which are central to personality development during adolescence. Participants rated statements such as “I enjoy trying new things” and “I like exploring new ideas” on a Likert scale ranging from “Strongly disagree” to “Strongly agree.” Higher scores reflected greater openness, a trait hypothesized to facilitate adolescents' receptiveness to unfamiliar artistic styles and cultural expressions.

Cognitive Flexibility: Cognitive Flexibility was measured using items adapted from a standardized Cognitive Flexibility Inventory, with wording adjusted to ensure clarity for adolescent respondents. These items assessed adolescents' perceived ability to adjust their thinking when encountering new information, alternative viewpoints, or unexpected situations (e.g., “I can change my way of thinking when I learn something new”).

Responses were recorded using the same Likert scale format as openness to experience. Higher scores indicated greater cognitive flexibility, a developmental capacity associated with adaptive learning, perspective-taking, and cross-cultural understanding during adolescence.

#### 3.2.5 Control variables

Several demographic variables were included as control variables to account for potential confounding influences on adolescents' aesthetic preferences. These included age (in years), gender, and current educational level (senior high school or first-year university). All control variables were measured via direct self-report items at the beginning of the questionnaire.

### 3.3 Validity and reliability of measures

Measurement reliability and validity were evaluated using the final sample data. Internal consistency reliability was first assessed using Cronbach's alpha and composite reliability (CR). All scales demonstrated acceptable to good reliability: aesthetic preference scales (α = 0.81–0.85), exposure to global art forms (α = 0.83), openness to experience (α = 0.79), and cognitive flexibility (α = 0.82). Composite reliability values for all constructs exceeded the recommended threshold of 0.70, indicating satisfactory internal consistency (Table 1).

**Table 1:** Reliability and convergent validity of study measures.

Construct	Cronbach's α	Composite reliability (CR)	Average variance extracted (AVE)	Standardized factor loadings
Chinese art preference	0.85	0.87	0.58	0.64–0.82
Western art preference	0.81	0.84	0.55	0.61–0.79
Exposure to global art forms	0.83	0.85	0.53	0.65–0.81
Openness to experience	0.79	0.82	0.51	0.62–0.77
Cognitive flexibility	0.82	0.84	0.54	0.64–0.80

All standardized factor loadings were statistically significant at *p* < 0.001. CR, Composite Reliability; AVE, Average Variance Extracted.

Construct validity was further examined through confirmatory factor analysis (CFA). The measurement model demonstrated acceptable fit to the data: χ^2^/df = 2.31, Comparative Fit Index (CFI) = 0.93, Tucker–Lewis Index (TLI) = 0.91, Root Mean Square Error of Approximation (RMSEA) = 0.058, and Standardized Root Mean Square Residual (SRMR) = 0.052. All standardized factor loadings exceeded 0.60 and were statistically significant (*p* < 0.001), supporting satisfactory construct validity (Table 2).

**Table 2:** Confirmatory factor analysis model fit indices.

Fit index	Recommended threshold	Obtained value
χ^2^/df	< 3.00	2.31
CFI	>0.90	0.93
TLI	>0.90	0.91
RMSEA	< 0.08	0.058
SRMR	< 0.08	0.052

Convergent validity was assessed using average variance extracted (AVE). AVE values for all constructs exceeded the recommended threshold of 0.50, indicating acceptable convergent validity. Discriminant validity was evaluated using the Fornell–Larcker criterion. Specifically, the square root of the AVE for each construct exceeded the correlations between constructs, supporting satisfactory discriminant validity among the study variables.

Because the present study relied primarily on self-report measures, Harman's single-factor test was conducted to assess the potential influence of common method variance (CMV). An exploratory factor analysis including all measurement items was performed without rotation. The results indicated that the first unrotated factor accounted for 28.46% of the total variance, which was below the commonly recommended threshold of 40%. These findings suggest that common method bias was unlikely to substantially threaten the validity of the present results.

These findings indicate that the measurement instruments demonstrated satisfactory psychometric properties and were appropriate for examining adolescents' cross-cultural aesthetic preferences and related developmental psychological constructs.

### 3.4 Data analysis

All statistical analyses were conducted using SPSS software. Data screening procedures were first applied to check for missing values, outliers, and normality assumptions. Descriptive statistics were computed to summarize adolescents' aesthetic preferences, cultural background distribution, exposure to global art forms, and psychological trait levels. Pearson correlation analyses were then performed to examine preliminary associations among the key variables. To test the study hypotheses, hierarchical linear regression analyses were conducted. Cultural background was entered as the predictor variable, aesthetic preferences as the outcome variable, and exposure to global art forms as the mediating variable. Openness to experience and cognitive flexibility were entered as moderating variables. Interaction terms were created by multiplying centered predictor and moderator variables to test moderation effects. These interaction terms were included in the regression models, and their statistical significance and effect sizes were examined to assess whether psychological traits moderated the relationship between cultural background and adolescents' aesthetic preferences. To further examine the indirect effect of exposure to global art forms, bootstrap mediation analyses were conducted using the PROCESS macro for SPSS (Model 4). Indirect effects were estimated using 5,000 bootstrap resamples with 95% bias-corrected confidence intervals. Mediation effects were considered statistically significant when the confidence intervals for the indirect effects did not include zero. Compared with traditional Sobel testing procedures, bootstrap mediation analysis provides improved robustness and statistical power for indirect effect estimation. Additional model diagnostics were conducted to assess multicollinearity, linearity, homoscedasticity, and residual normality assumptions. Variance Inflation Factor (VIF) values below the conventional threshold indicated no serious multicollinearity concerns among the predictor variables.

## 4 Results

### 4.1 Descriptive statistics

The final sample consisted of 400 adolescent participants, evenly distributed across cultural background groups, with 200 adolescents from Chinese cultural backgrounds and 200 from Western cultural backgrounds. All participants were aged between 14 and 18 years, corresponding to middle to late adolescence. The mean age of Chinese adolescents was 16.5 years (SD = 1.1), while the mean age of Western adolescents was 16.7 years (SD = 1.0), indicating comparable age distributions across groups. With respect to educational status, the majority of participants were senior high school students (Grades 10–12), while a smaller proportion were first-year university students. Educational composition was comparable between the two cultural groups, ensuring that differences in aesthetic preferences were not confounded by educational stage. Gender distribution was relatively balanced across groups, with male students accounting for approximately 45% of the Chinese group and 48% of the Western group, consistent with the overall sample composition.

Table 3 presents the means and standard deviations for the key study variables. Consistent with expectations, adolescents demonstrated culturally congruent aesthetic preferences. Adolescents from Chinese cultural backgrounds reported higher aesthetic preference ratings for Chinese artworks (M = 3.69, SD = 0.75) than for Western artworks (M = 3.12, SD = 0.69). Conversely, adolescents from Western cultural backgrounds showed stronger preferences for Western artworks (M = 3.57, SD = 0.72) than for Chinese artworks (M = 3.20, SD = 0.81). Across both groups, overall aesthetic preference levels were comparable, suggesting similar general engagement with visual art. Mean levels of openness to experience and cognitive flexibility were also similar between Chinese and Western adolescents, indicating comparable distributions of these developmental psychological traits across cultural contexts. However, Western adolescents reported higher levels of exposure to global art forms (M = 3.20, SD = 0.89) than their Chinese counterparts (M = 2.82, SD = 0.87), reflecting differences in access to or engagement with cross-cultural visual art during adolescence.

**Table 3:** Descriptive statistics for key variables by cultural background.

Variable	Chinese (*n =* 200)	Western (*n =* 200)
Chinese art preference, M (SD)	3.69 (0.75)	3.20 (0.81)
Western art preference, M (SD)	3.12 (0.69)	3.57 (0.72)
Overall aesthetic preference, M (SD)	3.40 (0.58)	3.39 (0.56)
Exposure to global art forms, M (SD)	2.82 (0.87)	3.20 (0.89)
Openness to experience, M (SD)	3.48 (0.82)	3.48 (0.79)
Cognitive flexibility, M (SD)	3.30 (0.71)	3.28 (0.69)

### 4.2 Correlation analysis

Pearson correlation analyses were conducted to examine the associations among cultural background, adolescents' aesthetic preferences, exposure to global art forms, and individual psychological traits. Table 4 presents the correlation matrix for the entire adolescent sample (*N* = 400). At the overall sample level, cultural background was significantly correlated with both Chinese art preference (*r* = −0.25, *p* < 0.001) and Western art preference (*r* = 0.27, *p* < 0.001). These results indicate that adolescents tended to show stronger aesthetic preferences for artworks originating from their own cultural tradition. In addition, exposure to global art forms was positively associated with cultural background (*r* = 0.20, *p* < 0.001), suggesting systematic differences in cross-cultural art engagement across cultural groups. Notably, openness to experience and cognitive flexibility were strongly and positively correlated with exposure to global art forms (*r* = 0.41, *p* < 0.001; *r* = 0.35, *p* < 0.001, respectively), as well as with each other (*r* = 0.47, *p* < 0.001). These associations are consistent with developmental theories suggesting that exploratory personality traits and adaptive cognitive capacities co-develop during adolescence and jointly support engagement with novel cultural content.

**Table 4:** Correlation matrix for key variables among adolescents (*N* = 400).

Variable	1	2	3	4	5	6
1. Cultural background	–					
2. Chinese art preference	−0.25^***^	–				
3. Western art preference	0.27^***^	−0.19^***^	–			
4. Exposure to global art forms	0.20^***^	−0.10^*^	0.03	–		
5. Openness to experience	0	−0.03	0.07	0.41^***^	–	
6. Cognitive flexibility	−0.01	−0.05	−0.04	0.35^***^	0.47^***^	–

^*^p < 0.05.

^***^p < 0.001.

To further clarify the role of exposure to global art forms in adolescents' aesthetic preferences, correlation analyses were conducted separately within each cultural background group. The results are summarized in Table 5. Among Chinese adolescents, exposure to global art forms was strongly negatively correlated with preference for Chinese artworks (*r* = −0.47, *p* < 0.001) and positively correlated with preference for Western artworks (*r* = 0.38, *p* < 0.001). A parallel pattern was observed among Western adolescents, for whom exposure to global art forms was negatively correlated with Western art preference (*r* = −0.46, *p* < 0.001) and positively correlated with Chinese art preference (*r* = 0.39, *p* < 0.001). These group-specific patterns suggest that greater exposure to global art during adolescence is associated with reduced preference for culturally familiar art and increased appreciation of culturally non-native artworks. This finding provides preliminary support for the proposed mediating role of global art exposure in the relationship between cultural background and adolescents' aesthetic preferences.

**Table 5:** Correlations between exposure to global art forms and aesthetic preferences within cultural groups.

Variable	Chinese adolescents (*n =* 200)	Western adolescents (*n =* 200)
Exposure to global art → chinese art preference	−0.47^***^	0.39^***^
Exposure to global art → western art preference	0.38^***^	−0.46^***^

^***^p < 0.001.

### 4.3 Hypothesis testing

#### 4.3.1 Cultural background and adolescents' aesthetic preferences

To test Hypothesis 1, hierarchical regression analyses were conducted to examine the relationship between cultural background and adolescents' aesthetic preferences, as well as the indirect role of exposure to global art forms. Separate regression models were estimated for Chinese art preference and Western art preference as outcome variables.

In Model 1, cultural background was entered as the sole predictor to assess its direct effect on adolescents' aesthetic preferences. As shown in Table 4, cultural background significantly predicted both Chinese art preference (β = −0.37, *p* < 0.001, *R*^2^ = 0.14) and Western art preference (β = 0.35, *p* < 0.001, *R*^2^ = 0.12). These results indicate that, during adolescence, individuals tended to show stronger aesthetic preferences for artworks originating from their own cultural tradition: Western adolescents rated Chinese artworks lower and Western artworks higher compared to their Chinese counterparts.

In Model 2, exposure to global art forms was added to the regression model to examine whether inclusion of this variable reduced the direct effect of cultural background on adolescents' aesthetic preferences. Exposure to global art forms significantly predicted both Chinese art preference (β = −0.25, *p* < 0.001) and Western art preference (β = 0.23, *p* < 0.001). Importantly, the inclusion of this variable reduced the magnitude of the cultural background effect on aesthetic preferences (Chinese art: β reduced from −0.37 to −0.30; Western art: β reduced from 0.35 to 0.28), suggesting a potential partial mediation effect (Table 6).

**Table 6:** Hierarchical regression analyses predicting adolescents' aesthetic preferences.

Variable	Chinese art preference			Western art preference		
	B	SE	β	B	SE	β
**Model 1**			*R^2^* = 0.14			*R^2^* = 0.12
Intercept	3.69	0.06		3.12	0.05	
Cultural background (Western)	−0.49	0.08	−0.37^***^	0.46	0.07	0.35^***^
**Model 2**			*R^2^* = 0.23			*R^2^* = 0.19
Intercept	4.32	0.13		2.58	0.12	
Cultural background (Western)	−0.39	0.08	−0.30^***^	0.37	0.07	0.28^***^
Exposure to global art forms	−0.22	0.04	−0.25^***^	0.19	0.04	0.23^***^
**Model 3**			*R^2^* = 0.28			*R^2^* = 0.24
Intercept	4.41	0.13		2.5	0.12	
Cultural background (Western)	−0.37	0.08	−0.28^***^	0.35	0.07	0.27^***^
Exposure to global art forms	−0.21	0.04	−0.24^***^	0.2	0.04	0.24^***^
Cultural background × exposure	0.34	0.06	0.22^***^	−0.31	0.06	−0.21^***^

^***^p < 0.001.

**Figure 2:**
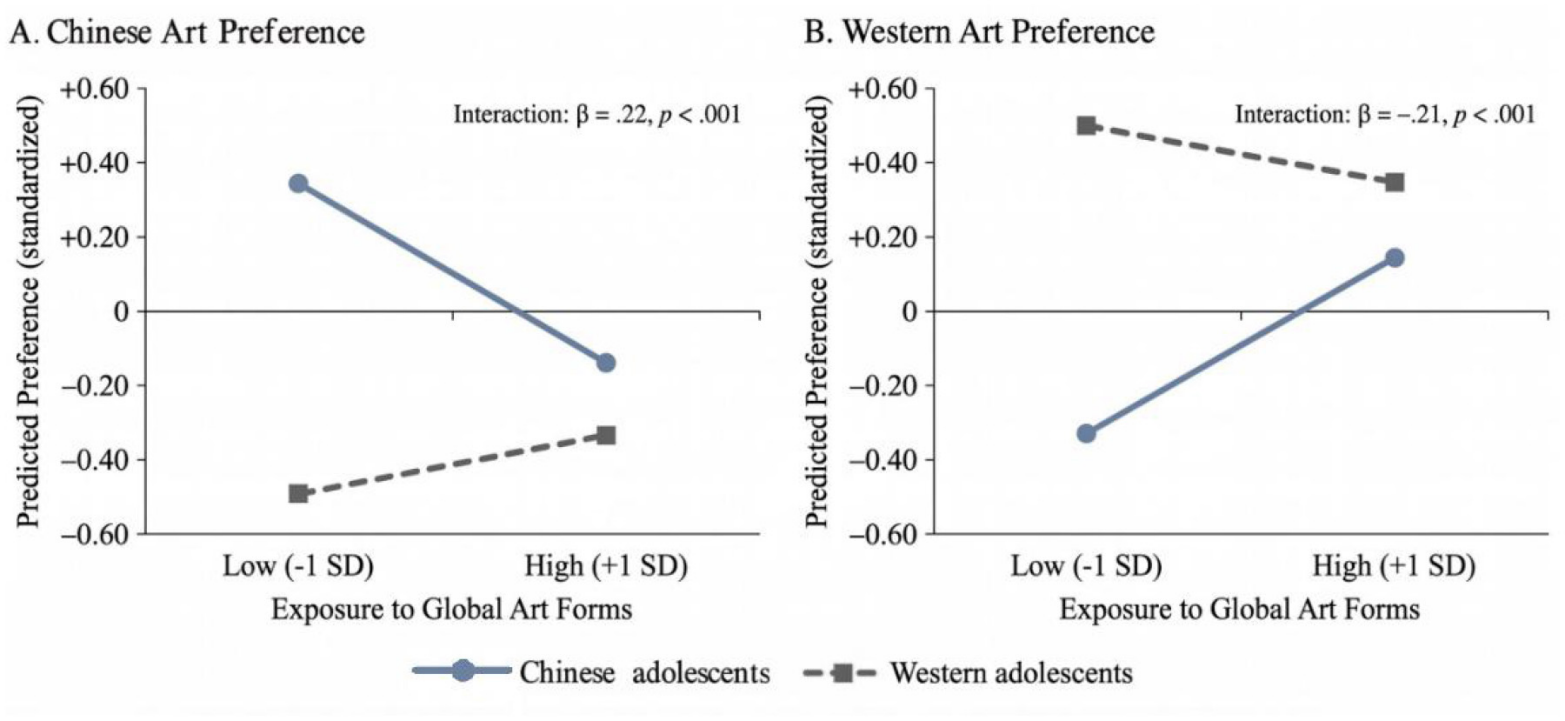
Predicted aesthetic preference differences across levels of exposure to global art forms. Lines represent predicted standardized preference scores at low and high levels of exposure to global art forms. **(A)** shows predicted Chinese art preference across levels of exposure to global art forms, whereas **(B)** shows predicted Western art preference across levels of exposure to global art forms.

To further examine whether exposure to global art forms interacted with cultural background, Model 3 included the interaction term between cultural background and exposure to global art forms. The interaction was significant for both Chinese art preference (β = 0.22, *p* < 0.001) and Western art preference (β = −0.21, *p* < 0.001), indicating that the strength of the relationship between cultural background and adolescents' aesthetic preferences varied according to adolescents' level of global art exposure. As illustrated in Figure 2, adolescents with lower exposure to global art forms showed stronger preferences for artworks associated with their own cultural tradition. In contrast, higher exposure to global art forms was associated with a reduced cultural gap in aesthetic preferences, suggesting that global artistic exposure may weaken culturally bounded aesthetic tendencies during adolescence.

To further examine the indirect effect of exposure to global art forms, bootstrap mediation analyses were conducted using the PROCESS macro with 5,000 bootstrap resamples and 95% bias-corrected confidence intervals. The results indicated that the indirect effects of cultural background on both Chinese art preference and Western art preference through exposure to global art forms were statistically significant, as the confidence intervals did not include zero.

Specifically, for Chinese art preference, the indirect effect was significant [indirect effect = −0.09, Boot SE = 0.03, 95% CI (−0.15, −0.04)]. Similarly, for Western art preference, the indirect effect was also significant [indirect effect = 0.08, Boot SE = 0.03, 95% CI (0.03, 0.14)]. These findings provide robust support for the partial mediating role of exposure to global art forms in the relationship between cultural background and adolescents' aesthetic preferences (Table 7).

**Table 7:** Bootstrap mediation analysis of exposure to global art forms.

Outcome variable	Indirect effect	Boot SE	95% CI lower	95% CI upper
Chinese art preference	−0.09	0.03	−0.15	−0.04
Western art preference	0.08	0.03	0.03	0.14

Bootstrap mediation analyses were conducted using 5,000 bootstrap resamples and 95% bias-corrected confidence intervals. Indirect effects were considered statistically significant when the confidence interval did not include zero.

#### 4.3.2 Moderating role of openness to experience in adolescents

**Figure 3:**
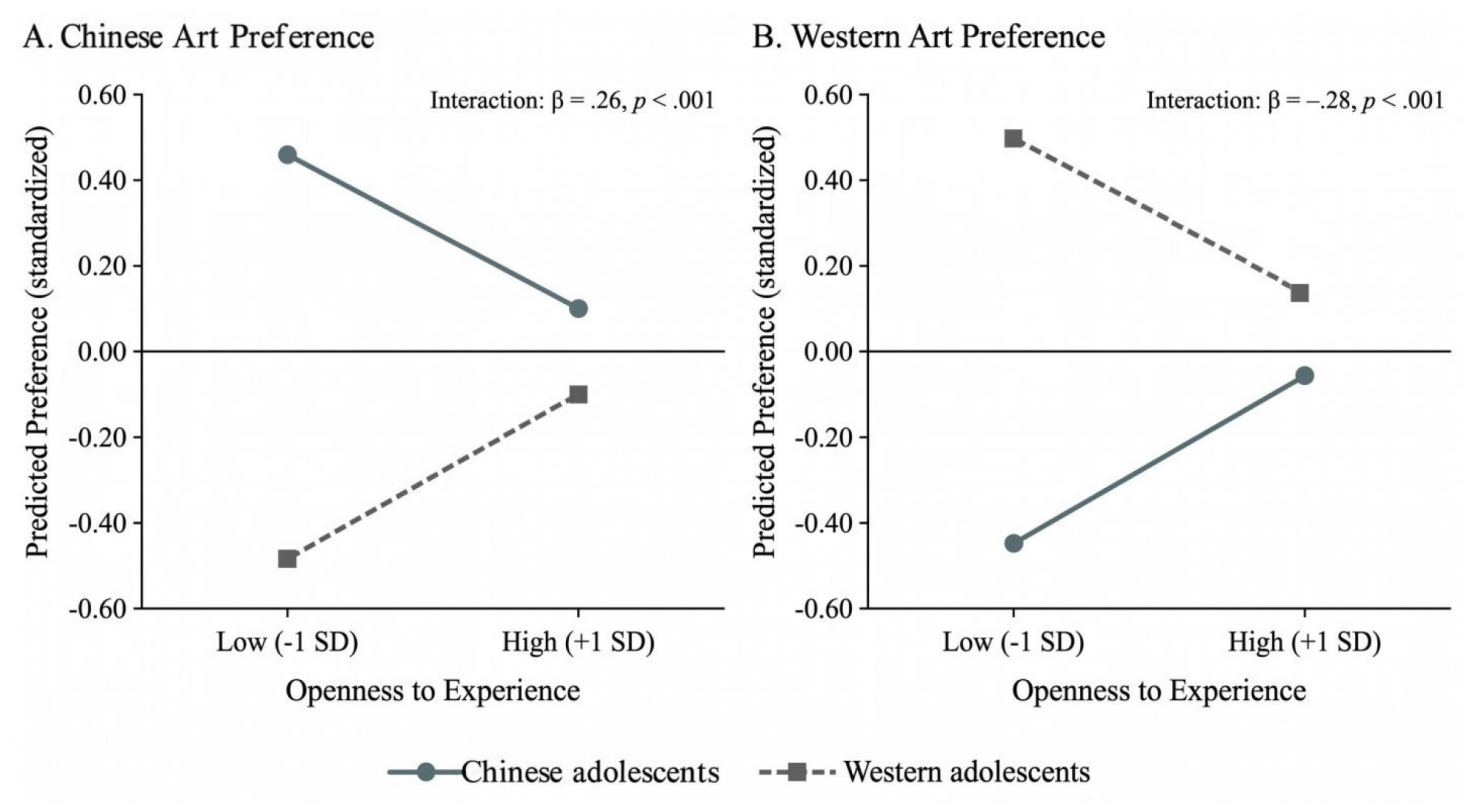
Moderating role of openness to experience in adolescents' aesthetic preferences.Note. Lines represent predicted standardized preference scores at low and high levels of openness to experience. **(A)** shows the moderating effect of openness to experience on Chinese art preference, whereas **(B)** shows the moderating effect of openness to experience on Western art preference.

To test Hypothesis 2, moderation analyses were conducted to examine whether openness to experience, a key personality trait undergoing active development during adolescence, moderated the relationship between cultural background and adolescents' aesthetic preferences. Separate regression models were estimated for Chinese and Western art preference outcomes, with cultural background, openness to experience, and their interaction entered simultaneously. As shown in Table 8, the interaction between cultural background and openness to experience was significant for both Chinese art preference (β = 0.26, *p* < 0.001) and Western art preference (β = −0.28, *p* < 0.001). These findings indicate that openness to experience significantly moderates the association between cultural background and adolescents' aesthetic preferences. As illustrated in Figure 3, adolescents lower in openness to experience demonstrated stronger culturally congruent aesthetic preferences, showing greater preference for artworks associated with their own cultural background. In contrast, adolescents higher in openness to experience exhibited a reduced cultural preference gap across both Chinese and Western art evaluations.

**Table 8:** Moderation analysis: openness to experience predicting adolescents' aesthetic preferences.

Variable	Chinese art preference			Western art preference		
	B	SE	β	B	SE	β
Intercept	3.7	0.05		3.11	0.05	
Cultural background (Western)	−0.48	0.07	−0.36^***^	0.44	0.07	0.33^***^
Openness to experience	−0.17	0.04	−0.19^***^	0.14	0.04	0.16^***^
Cultural background × openness	0.41	0.07	0.26^***^	−0.43	0.07	−0.28^***^
*R^2^*			0.25			0.24

^***^p < 0.001.

Specifically, adolescents higher in openness to experience exhibited weaker culturally congruent preference patterns compared to those lower in openness. These interaction patterns suggest that openness to experience attenuates the influence of cultural background on adolescents' aesthetic judgments. This suggests that, during adolescence, a greater tendency toward curiosity and exploration attenuates the influence of culturally ingrained aesthetic schemas. More broadly, these findings imply that openness to experience may facilitate greater aesthetic flexibility and reduce reliance on culturally shaped evaluative schemas during adolescence (see Figure 3).

Importantly, although the observed interaction effects were moderate in statistical magnitude, the moderation patterns nonetheless reflect meaningful developmental differences during adolescence. Adolescents with higher openness to experience demonstrated noticeably greater responsiveness toward culturally unfamiliar artworks and reduced dependence on culturally familiar evaluative frameworks. From a developmental psychology perspective, these effects are meaningful because adolescence represents a period of heightened cognitive-emotional plasticity, identity exploration, and expanding cultural learning experiences. Consequently, even moderate differences in openness to experience may substantially influence how adolescents interpret, evaluate, and emotionally engage with culturally diverse visual stimuli. The findings therefore suggest that openness to experience is not merely a statistically significant moderator, but also a developmentally relevant psychological characteristic shaping adolescents' cross-cultural aesthetic engagement.

#### 4.3.3 Moderating role of cognitive flexibility in adolescents

**Figure 4:**
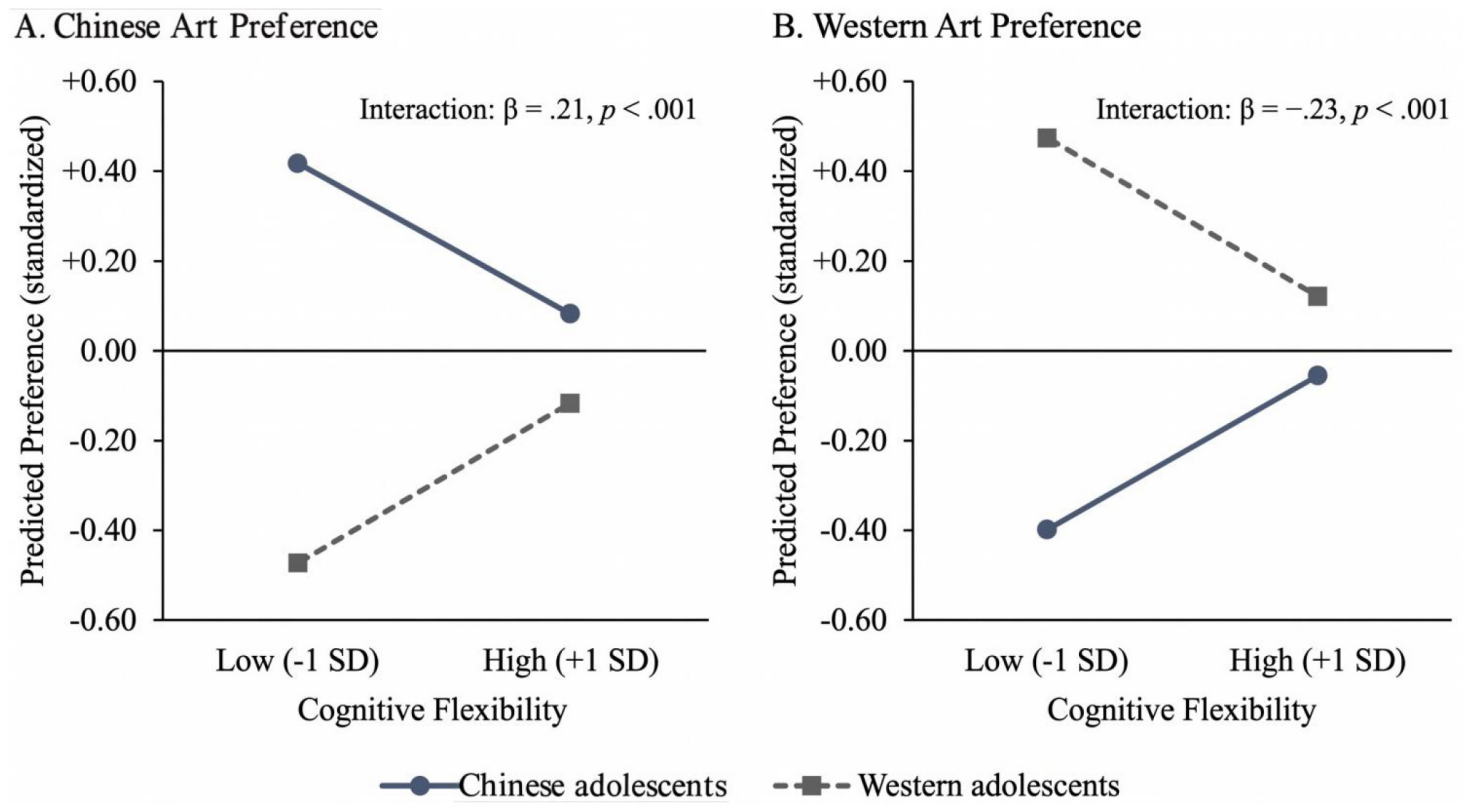
Moderating role of cognitive flexibility in adolescents' aesthetic preferences. Lines represent predicted standardized preference scores at low and high levels of cognitive flexibility. **(A)** shows the moderating effect of cognitive flexibility on Chinese art preference, whereas **(B)** shows the moderating effect of cognitive flexibility on Western art preference.

To test Hypothesis 3, moderation analyses were conducted to examine whether cognitive flexibility, a core component of executive functioning that continues to develop throughout adolescence, moderated the relationship between cultural background and adolescents' aesthetic preferences. Separate regression models were estimated for Chinese and Western art preference outcomes, with cultural background, cognitive flexibility, and their interaction entered simultaneously. As shown in Table 9, the interaction between cultural background and cognitive flexibility was significant for both Chinese art preference (β = 0.21, *p* < 0.001) and Western art preference (β = −0.23, *p* < 0.001). These findings indicate that cognitive flexibility significantly moderates the association between cultural background and adolescents' aesthetic preferences. As illustrated in Figure 4, adolescents with lower cognitive flexibility demonstrated stronger culturally congruent aesthetic preferences, showing greater preference for artworks associated with their own cultural background. In contrast, adolescents with higher cognitive flexibility exhibited a reduced cultural preference gap across both Chinese and Western art evaluations. Specifically, adolescents with higher cognitive flexibility exhibited weaker culturally congruent aesthetic preferences than those with lower cognitive flexibility. These interaction patterns suggest that cognitive flexibility attenuates the influence of cultural background on adolescents' aesthetic judgments by promoting more adaptive and flexible evaluative processing. This pattern suggests that, during adolescence, greater capacity for adaptive thinking and perspective shifting reduces reliance on culturally familiar aesthetic frameworks when evaluating visual art. More broadly, these findings imply that cognitive flexibility may facilitate greater openness to diverse aesthetic experiences and reduce reliance on culturally ingrained evaluative schemas during adolescence (see Figure 4).

**Table 9:** Moderation analysis: cognitive flexibility predicting adolescents' aesthetic preferences.

Variable	Chinese art preference			Western art preference		
	B	SE	β	B	SE	β
Intercept	3.69	0.05		3.12	0.05	
Cultural background (Western)	−0.48	0.07	−0.37^***^	0.45	0.07	0.34^***^
Cognitive flexibility	−0.14	0.04	−0.16^**^	0.11	0.04	0.13^**^
Cultural background × cognitive flexibility	0.35	0.08	0.21^***^	−0.38	0.08	−0.23^***^
*R^2^*			0.22			0.2

^**^p < 0.01.

^***^p < 0.001.

Importantly, adolescents with higher cognitive flexibility demonstrated noticeably greater willingness to engage with culturally unfamiliar artworks and reduced dependence on culturally familiar evaluative schemas compared to adolescents with lower cognitive flexibility. From a developmental perspective, these findings are meaningful because cognitive flexibility is closely associated with executive functioning, adaptive thinking, and perspective shifting processes that continue to mature throughout adolescence. Consequently, even moderate differences in cognitive flexibility may substantially influence adolescents' capacity to interpret and evaluate culturally diverse visual stimuli in more adaptive and less culturally constrained ways. The findings therefore suggest that cognitive flexibility represents not only a statistically significant moderator, but also a developmentally important psychological characteristic contributing to adolescents' cross-cultural aesthetic responsiveness and evaluative adaptability.

#### 4.3.4 Developmental differences across adolescent subgroups

To further examine whether developmental stage influenced adolescents' cross-cultural aesthetic preferences, supplementary subgroup analyses were conducted across different stages of adolescence. Following commonly used developmental psychology classifications, participants were grouped into early adolescence (14–15 years), middle adolescence (16–17 years), and late adolescence (18 years). One-way ANOVA procedures were conducted to compare aesthetic preferences, exposure to global art forms, openness to experience, and cognitive flexibility across these developmental subgroups.

As shown in Table 10, significant developmental differences emerged across several variables. Exposure to global art forms increased progressively across developmental stages, with late adolescents reporting significantly higher levels of global artistic exposure compared to early adolescents (*F* = 8.17, *p* < 0.001). Similarly, openness to experience differed significantly across age groups (*F* = 5.24, *p* < 0.01), with late adolescents demonstrating greater openness to culturally unfamiliar artistic content.

**Table 10:** Developmental differences across adolescent subgroups.

Variable	Early adolescence (14–15) mean (SD)	Middle adolescence (16–17) mean (SD)	Late adolescence (18) mean (SD)	F	*p*
Chinese art preference	4.26 (0.68)	4.09 (0.66)	3.93 (0.64)	4.11	0.017^*^
Western art preference	3.18 (0.72)	3.36 (0.70)	3.55 (0.67)	4.87	0.009^**^
Exposure to global art forms	3.14 (0.73)	3.45 (0.71)	3.77 (0.69)	8.17	< 0.001^***^
Openness to experience	3.42 (0.62)	3.57 (0.60)	3.74 (0.58)	5.24	0.006^**^
Cognitive flexibility	3.36 (0.59)	3.50 (0.57)	3.70 (0.55)	5.96	0.003^**^

One-way ANOVA analyses were conducted to examine developmental subgroup differences across adolescence. Early adolescence = 14–15 years; middle adolescence = 16–17 years; late adolescence = 18 years.

^*^p < 0.05.

^**^p < 0.01.

^***^p < 0.001.

For aesthetic preference outcomes, early adolescents exhibited stronger culturally congruent aesthetic preferences, showing relatively higher preference for artworks associated with their own cultural background. In contrast, late adolescents demonstrated comparatively reduced cultural preference differences across both Chinese and Western art evaluations. Specifically, Chinese art preference scores gradually decreased across developmental stages, whereas Western art preference scores showed a moderate increase among older adolescents. These findings suggest that older adolescents may evaluate culturally unfamiliar artworks in a more flexible and less culturally bounded manner.

Cognitive flexibility also showed significant developmental variation across adolescent stages (*F* = 5.96, *p* < 0.01). Late adolescents reported higher levels of cognitive flexibility than early adolescents, suggesting developmental increases in adaptive thinking and perspective shifting during later stages of adolescence.

Overall, these subgroup analyses provide supplementary developmental evidence supporting the proposition that cross-cultural aesthetic preferences become progressively more flexible and less culturally constrained across adolescence. However, because the present study employed a cross-sectional rather than longitudinal design, these findings should be interpreted as age-related developmental differences rather than direct developmental trajectories.

### 4.4 Integrative multivariable analysis of adolescents' cross-cultural aesthetic preferences

Rather than testing a hypothesis in the strict inferential sense, this analysis evaluated whether integrative multivariable regression models incorporating cultural background, exposure to global art forms, and individual psychological traits explained additional variance in adolescents' aesthetic preferences beyond single-factor models. Hypothesis 4 proposed that considering multiple predictors jointly could provide a more comprehensive understanding of cross-cultural aesthetic preferences compared to single-factor models. To examine this proposition, a series of hierarchical regression models with progressively integrated predictor variables were estimated, as summarized in Table 11.

**Table 11:** Hierarchical multivariable regression models predicting adolescents' aesthetic preferences.

Model	Predictors included	Chinese art preference (*R^2^*)	Western art preference (*R^2^*)
Model A	Cultural background only	0.14	0.12
Model B	Cultural background + exposure to global art forms	0.23	0.19
Model C	Model B + openness to experience	0.25	0.24
Model D	Model B + cognitive flexibility	0.22	0.2
Model E	Full model (culture + exposure + openness + cognitive flexibility + interaction terms)	0.28	0.24

As shown in Table 8, models including cultural background alone explained a limited proportion of variance in adolescents' aesthetic preferences (Chinese art preference: *R*^2^ = 0.14; Western art preference: *R*^2^ = 0.12). While these results confirm the importance of cultural background, they also suggest that culture alone provides an incomplete account of adolescents' aesthetic development. When exposure to global art forms was added to the model, the explained variance increased for both Chinese and Western art preferences (Chinese art preference: *R*^2^ = 0.23; Western art preference: *R*^2^ = 0.19). This improvement highlights the contribution of learning-related exposure in shaping adolescents' aesthetic preferences.

Further increases in explained variance were observed when individual psychological traits were incorporated. Models including openness to experience and cognitive flexibility accounted for additional variance in aesthetic preferences, reflecting the contribution of developmental characteristics related to exploration, adaptability, and executive functioning. Notably, the inclusion of these traits allowed the models to capture individual differences in how adolescents respond to culturally familiar vs. unfamiliar artworks. The full multivariable model, integrating cultural background, exposure to global art forms, openness to experience, cognitive flexibility, and their interaction terms, yielded the highest explanatory power for both outcome variables (Chinese art preference: *R*^2^ = 0.28; Western art preference: *R*^2^ = 0.24). These results should be interpreted as providing preliminary evidence of the incremental explanatory value of multivariable regression models rather than as a formal test of Hypothesis 4.

Additional model diagnostics were conducted to ensure robustness. Variance Inflation Factor (VIF) values for all predictors were below 2.45, indicating no serious multicollinearity concerns. Residuals were examined to confirm the assumptions of linearity, homoscedasticity, and approximate normality. Standardized regression coefficients are reported to facilitate effect size interpretation.

## 5 Discussion

The present study examined how cultural background, exposure to global art forms, and individual psychological traits jointly shape adolescents' aesthetic preferences in visual arts. By focusing on adolescents aged 14–18 and adopting a multivariable analytical framework, this research contributes to a developmentally grounded understanding of cross-cultural aesthetic engagement within broader contemporary sociocultural and media contexts. Rather than treating aesthetic preferences as static outcomes of cultural membership, the findings highlight the importance of learning-related exposure and individual developmental characteristics in shaping adolescents' responses to visual art. At the same time, the present study conceptualizes contemporary media and digital environments primarily as broader contextual conditions that may expand adolescents' opportunities for cross-cultural artistic exposure, rather than as directly measured explanatory variables within the empirical model. The findings therefore should be interpreted as reflecting the joint influence of developmental cultural socialization, exposure to diverse artistic traditions, and individual psychological characteristics on adolescents' aesthetic preferences.

### 5.1 Cultural influences on adolescents' aesthetic preferences

Consistent with previous cross-cultural research, the results indicate that cultural background remains a significant predictor of aesthetic preferences during adolescence, with adolescents generally showing stronger appreciation for artworks originating from their own cultural tradition. This pattern aligns with earlier findings suggesting that aesthetic judgments are associated with culturally embedded perceptual habits, symbolic systems, and artistic conventions acquired through socialization (Masuda et al., 2008).

From a developmental perspective, such culturally congruent preferences can be understood as reflecting the internalization of cultural cognitive schemas during adolescence. Chinese adolescents' higher appreciation for Chinese artworks may be related to aesthetic principles emphasizing harmony, balance, and relational context, which are central to many Chinese artistic traditions and educational practices (Wang et al., 2023). Similarly, Western adolescents' stronger preference for Western art likely reflects greater familiarity with aesthetic values emphasizing realism, individual expression, and emotional salience that characterize much of Western art history (Chatterjee and Vartanian, 2014). Importantly, these preferences do not imply fixed or essentialized cultural differences, but rather reflect dominant learning environments and exposure patterns during a formative developmental period.

Beyond documenting cultural differences, the present findings extend prior research by examining the potential indirect role through which cultural background may be associated with adolescents' aesthetic preferences. The observed indirect association involving exposure to global art forms suggests that adolescents' engagement with culturally diverse visual art—particularly within broader digital and globalized media contexts—may partially account for variations in culturally congruent aesthetic preferences. This finding supports the view that aesthetic development during adolescence is dynamic and potentially responsive to learning opportunities afforded by contemporary cross-cultural environments (Swami, 2013). However, because the present study employed a cross-sectional design, these findings should be interpreted as correlational associations rather than evidence of directional developmental influence or causal mediation processes.

The incremental variance explained by exposure to global art forms, as demonstrated in the multivariable models summarized in Table 11, suggests that cultural background alone provides an incomplete account of adolescents' aesthetic development. As adolescents report greater exposure to artworks from different cultural traditions, the predictive association between cultural background and aesthetic preference becomes comparatively weaker, indicating greater openness toward non-native aesthetic forms. This finding resonates with prior work suggesting that cross-cultural exposure may be associated with broader aesthetic appreciation and reduced culturally bound evaluative biases (van Paasschen et al., 2015). At the same time, the indirect associations observed in the present regression-based analyses were partial rather than fully explanatory, indicating that cultural background continued to demonstrate meaningful associations with adolescents' aesthetic judgments even after accounting for exposure-related variables. Accordingly, the present findings should be interpreted as preliminary developmental evidence of indirect cross-cultural learning associations rather than definitive causal mediation effects.

### 5.2 Individual psychological traits and developmental differences in aesthetic engagement

A central contribution of this study lies in demonstrating that individual psychological traits—specifically openness to experience and cognitive flexibility—moderate the relationship between cultural background and adolescents' aesthetic preferences. These findings underscore the importance of considering individual developmental differences when examining cross-cultural aesthetics, particularly during adolescence, a period marked by substantial variability in personality development and executive functioning.

Adolescents higher in openness to experience exhibited reduced cultural bias in their aesthetic preferences, showing greater appreciation for artworks from cultures other than their own. This pattern is consistent with previous research linking openness to curiosity, imagination, and receptivity to novelty (Fayn et al., 2015), and suggests that adolescents who are more inclined toward exploration may be more willing to engage with unfamiliar aesthetic systems. Rather than rejecting culturally unfamiliar art, these adolescents may approach it as an opportunity for learning and meaning-making, thereby reducing the relative strength of culturally ingrained preference patterns (Swami, 2013).

Cognitive flexibility similarly emerged as a significant moderator, with adolescents higher in this trait displaying less culturally constrained aesthetic preferences. Cognitive flexibility, as a component of executive functioning that continues to mature throughout adolescence, supports the ability to shift perspectives, integrate multiple sources of information, and adapt interpretive strategies (Cox et al., 2025). In the context of art appreciation, cognitively flexible adolescents may be better equipped to interpret unfamiliar visual symbols and compositional principles without relying solely on culturally familiar evaluative frameworks. Together, these moderating effects highlight that adolescents' aesthetic preferences are associated with interactions between cultural learning environments and individual developmental capacities. While cultural background may provide an initial framework for aesthetic evaluation, personality traits and cognitive skills may relate to how flexibly adolescents apply or revise these frameworks when encountering culturally diverse art. Importantly, the present findings should not be interpreted as evidence of causal developmental influence because the cross-sectional design does not permit conclusions regarding temporal directionality or developmental change over time. Longitudinal and experimental research designs would be necessary to more directly examine causal developmental mechanisms underlying adolescents' cross-cultural aesthetic engagement.

## 6 Conclusion and limitations

### 6.1 Conclusion

This study examined adolescents' aesthetic preferences in visual arts from a developmental and cross-cultural perspective, focusing on how cultural background, exposure to global art forms, and individual psychological traits jointly shape aesthetic judgments during adolescence. The findings demonstrate that cultural background remains an important factor in adolescents' aesthetic preferences, with a tendency to favor artworks from one's own cultural tradition. However, this influence is neither fixed nor deterministic. Exposure to global art forms partially mediated the relationship between cultural background and aesthetic preferences, indicating that adolescents' aesthetic development is responsive to learning experiences afforded by digital and globalized visual environments. Moreover, openness to experience and cognitive flexibility moderated cultural effects, such that adolescents higher in these traits showed reduced cultural bias in aesthetic judgments. Taken together, and supported by the incremental explanatory power of multivariable models, the results suggest that adolescents' cross-cultural aesthetic preferences are best understood as the outcome of interacting cultural, experiential, and developmental factors rather than cultural background alone. By integrating cultural psychology, developmental psychology, and data-informed analytical approaches, this study contributes to a more nuanced understanding of aesthetic development in adolescence within contemporary digital contexts.

### 6.2 Limitations

Despite these contributions, several limitations should be acknowledged. First, although the sample was developmentally focused and culturally balanced, cultural background was operationalized using broad Chinese–Western categories, which may overlook meaningful variation within cultural groups. In particular, the Western cultural background group included adolescents from diverse North American and European family and educational backgrounds, and the present classification approach cannot fully capture the complexity of individual cultural identity, acculturation processes, or multicultural experiences. The categorization strategy was intended to reflect dominant developmental cultural environments rather than culturally homogeneous groups. Moreover, substantial heterogeneity may exist within both Chinese and Western cultural categories, including differences related to ethnicity, regional cultural traditions, language use, bilingual or multilingual experiences, educational systems, and varying levels of engagement with globalized media environments. Such factors may influence adolescents' aesthetic preferences in ways that were not fully captured in the present study. Therefore, the findings should be interpreted within the context of broad developmental cultural socialization environments rather than as reflecting fixed or essentialized cultural identities. Second, the cross-sectional design limits inferences about developmental change over time; longitudinal research would be necessary to examine how adolescents' aesthetic preferences evolve with continued exposure to global art and maturation of psychological traits. Third, the reliance on self-report measures may introduce response biases and cannot fully capture the complexity of adolescents' real-time aesthetic experiences in digital environments. Although Harman's single-factor test suggested that common method variance was unlikely to substantially threaten the validity of the findings, the present study relied primarily on self-report measures collected at a single time point. Therefore, potential social desirability effects and shared method variance cannot be completely ruled out. Future research should incorporate multi-method approaches, behavioral measures, observational methods, or longitudinal designs to further reduce potential common method bias and improve methodological robustness. Fourth, potential confounding variables, including socioeconomic status, prior formal art education, intensity of digital media usage, urban vs. rural context, and previous international exposure, were not fully controlled in the present study. These factors may partially explain differences attributed to cultural background and should be considered in future research to more precisely isolate the effects of cultural and developmental variables. In addition, several contextual educational and family-related variables that may meaningfully relate to adolescents' aesthetic preferences were not directly measured or statistically controlled in the present study. These variables include differences in formal art training, school curriculum structure, access to arts education resources, parental cultural capital, and broader socioeconomic conditions associated with exposure to cultural activities and artistic learning opportunities. Because adolescents' aesthetic experiences are often shaped by both school-based and family-based cultural environments, these unmeasured contextual factors may partially contribute to the observed cross-cultural differences in aesthetic preferences. Future research should incorporate more detailed educational and family-context variables in order to better distinguish the relative contributions of cultural background, developmental psychological traits, and sociocultural learning environments to adolescents' cross-cultural aesthetic engagement. Fifth, future research should incorporate digital behavioral data, computational methods, and large-scale multimodal datasets to more fully realize the broader data-informed perspectives discussed in the theoretical background. Finally, future studies should also employ more fine-grained measures of cultural identity and cross-cultural experience, including acculturation, bilingualism, and multicultural educational exposure, to better account for within-group cultural diversity among adolescents. Addressing these limitations would further strengthen the developmental and ecological validity of research on cross-cultural aesthetic appreciation among adolescents.

## Data Availability

The original contributions presented in the study are included in the article/Supplementary material, further inquiries can be directed to the corresponding author.
