# Supplementary material for: Adolescents' cross-cultural aesthetic preferences are shaped by cultural background, art exposure, and developmental psychological traits
Source: Front Psychol. 2026 Jun 18;17:1774986. doi: 10.3389/fpsyg.2026.1774986 (PMC13322890; doi:10.3389/fpsyg.2026.1774986)

Supplementary Figure S1. Representative examples of visual artworks used in the aesthetic preference task.

All images were obtained from public-domain or open-access museum collections for academic research purposes.

(A) Chinese landscape painting


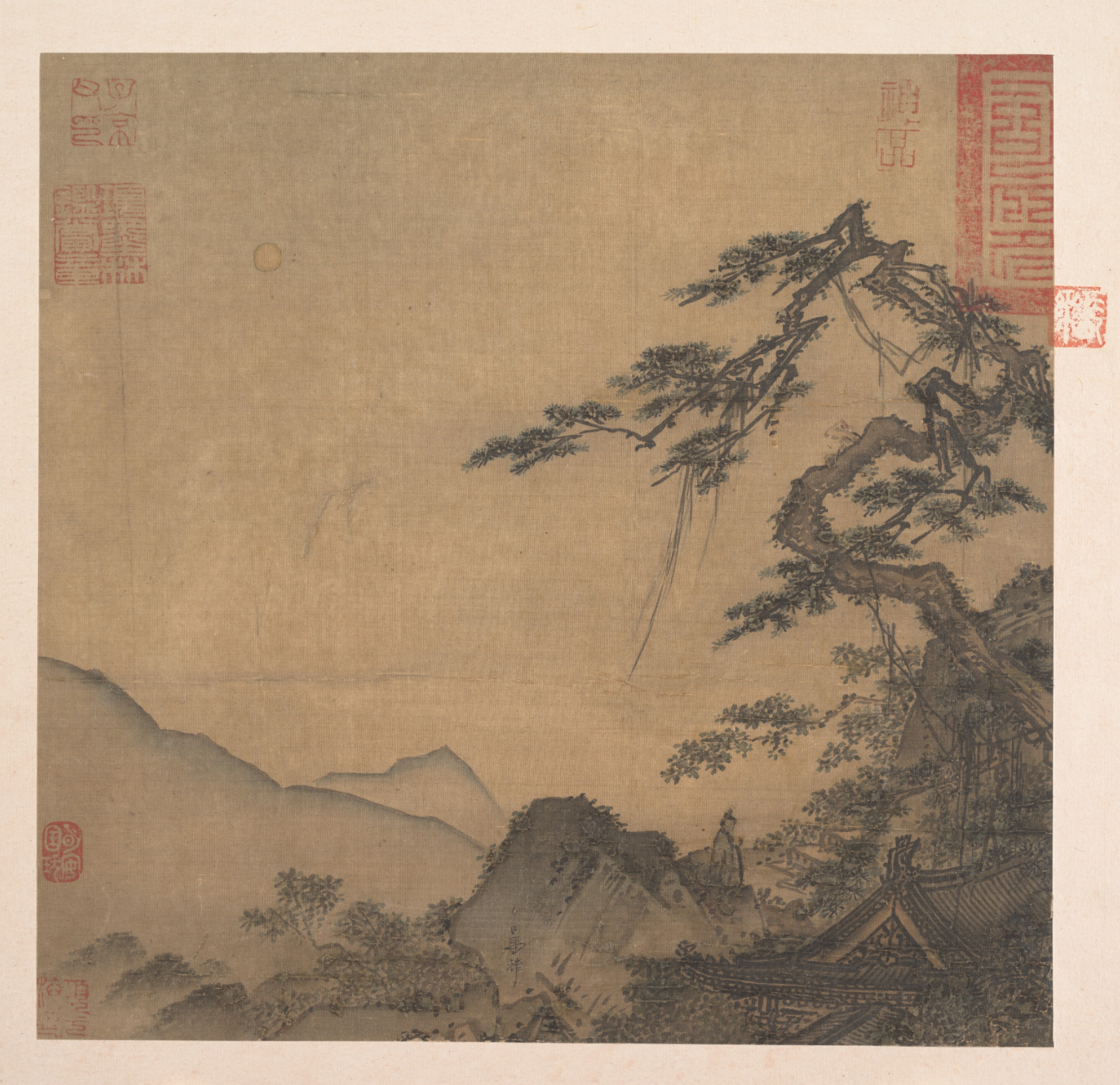


(B) Chinese ink bamboo painting


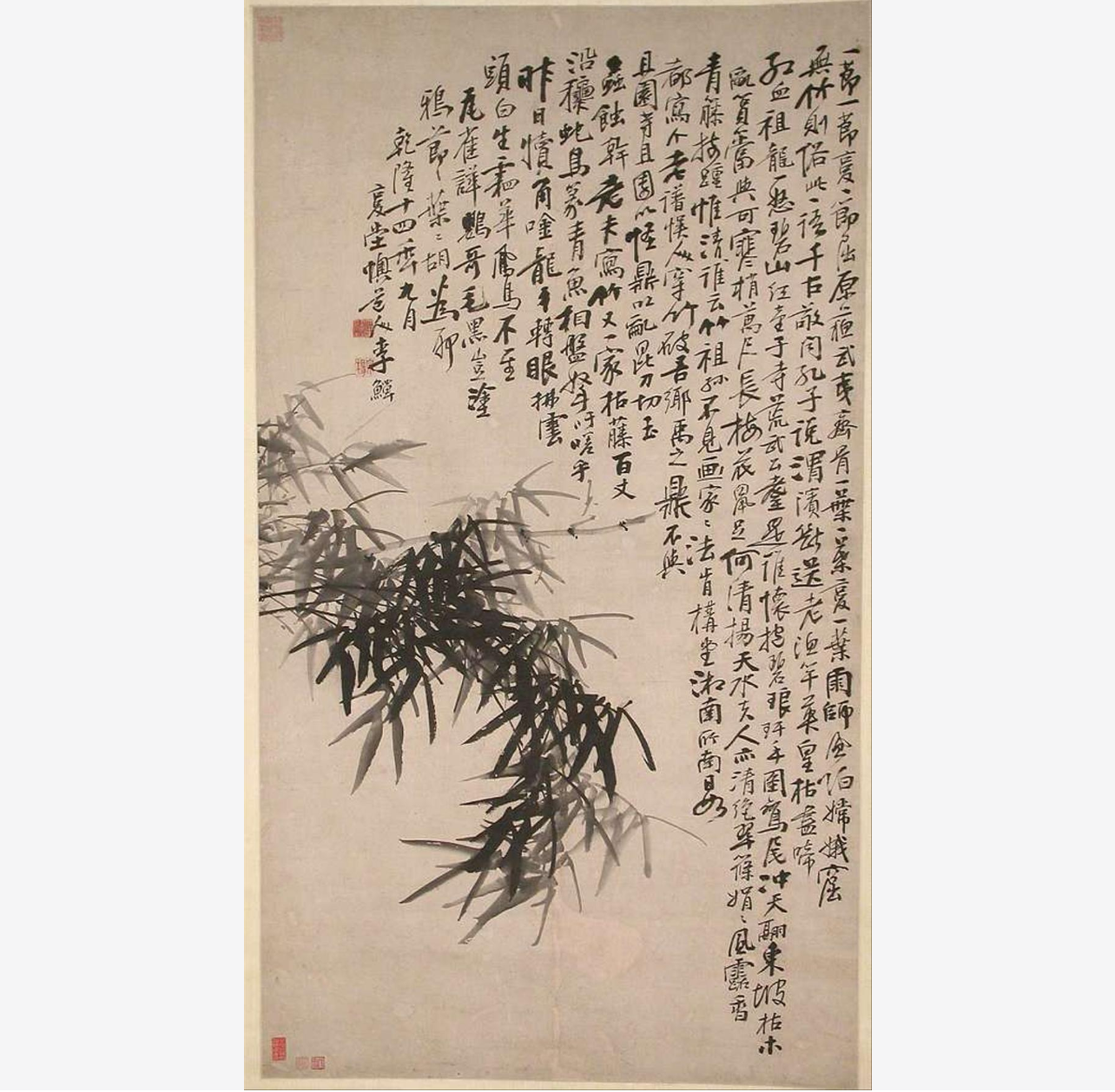


(C) Claude Monet, Water Lilies


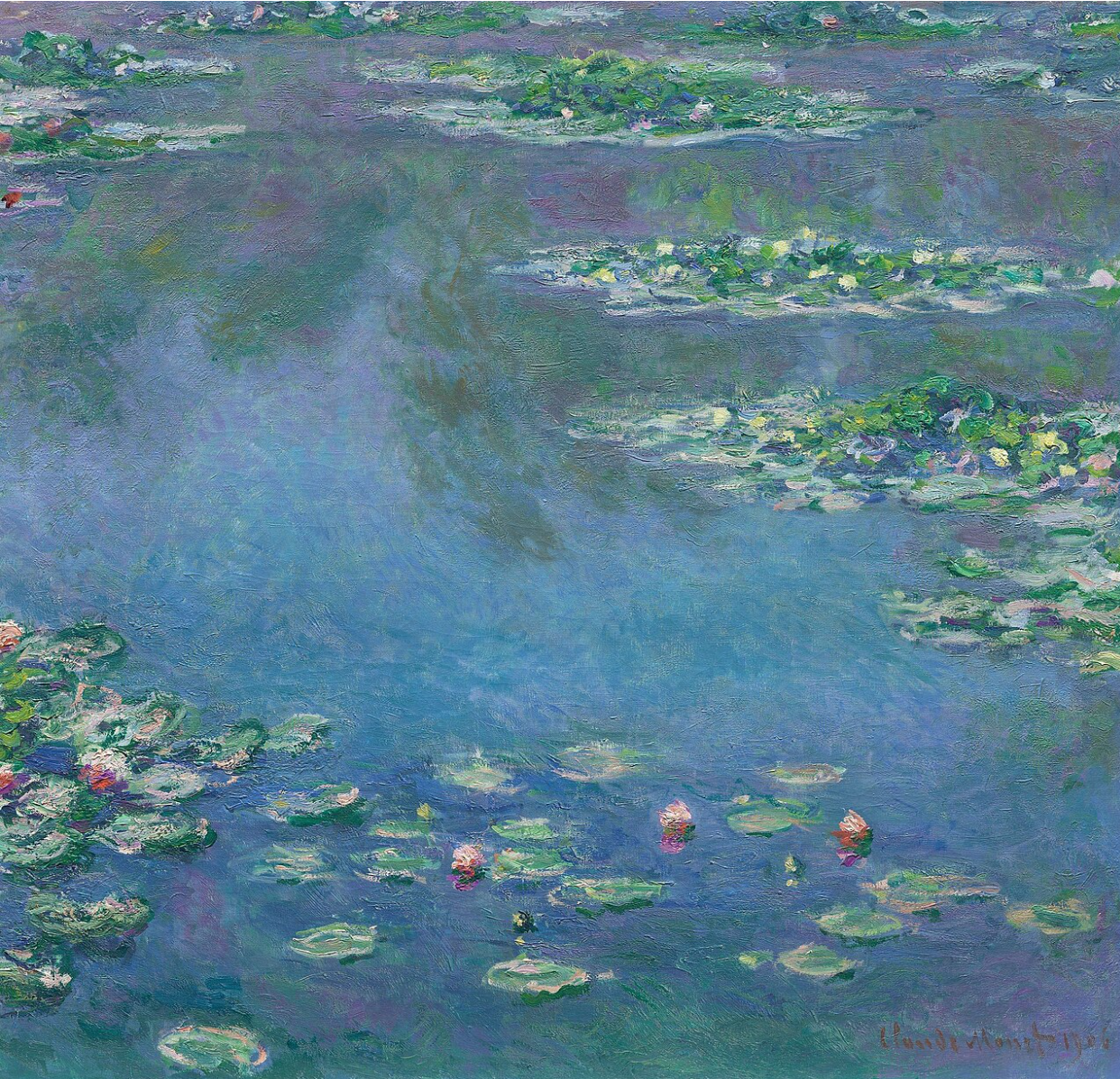


(D) Claude Monet, The Magpie


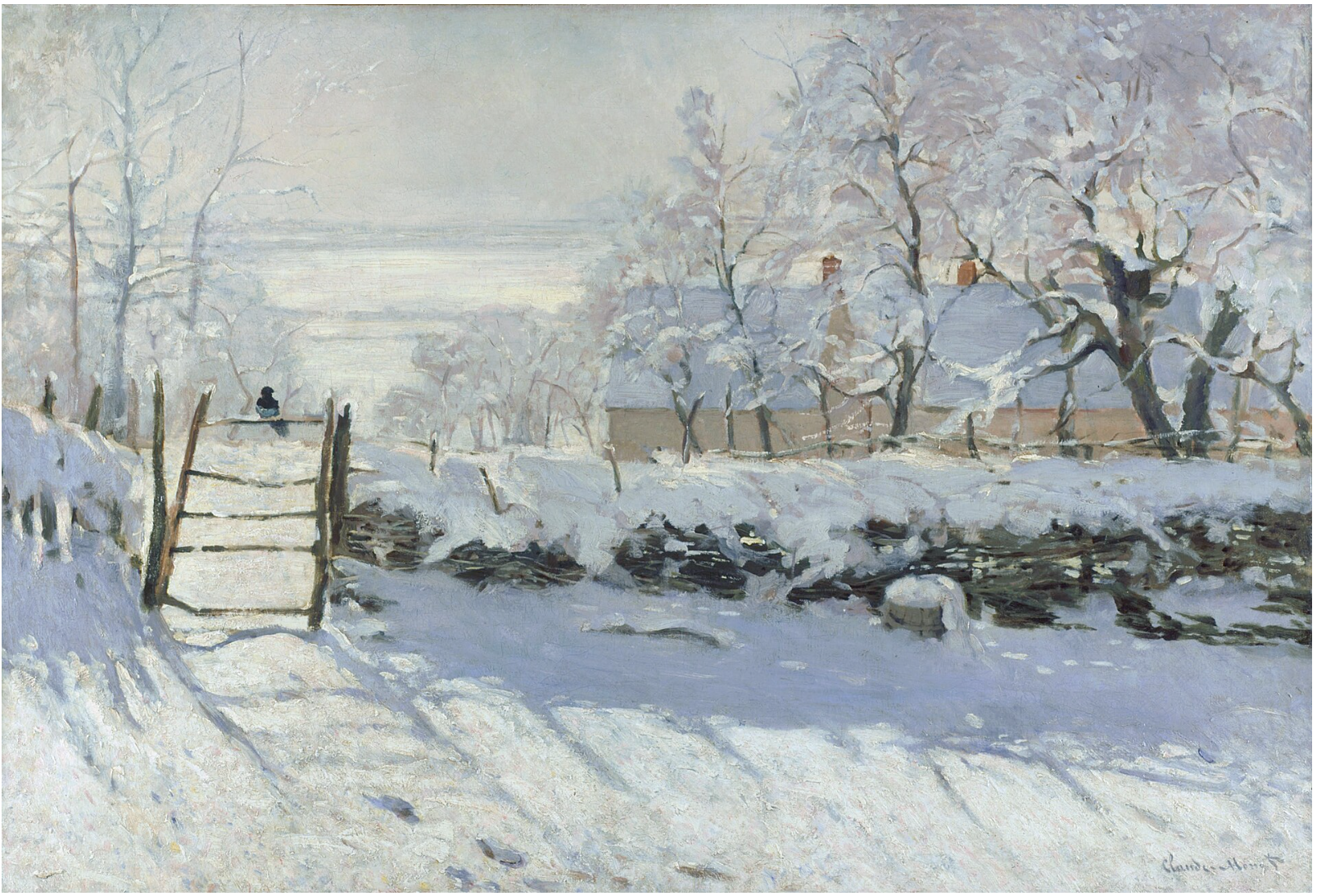

Supplement: Supplementary file 1 [file Data_Sheet_1.docx]
